# Supplementary material for: The type of diet consumed during prepuberty modulates plasma cholesterol, hepatic LXRα expression, and DNA methylation and hydroxymethylation during adulthood in male rats
Source: PLoS One. 2025 Jan 24;20(1):e0315197. doi: 10.1371/journal.pone.0315197 (PMC11761095; doi:10.1371/journal.pone.0315197)
Supplement: S1 Fig — Values are means ± SEMs (n = 5–6 rats per group). ANOVA analyses were performed followed by Tukey´s post hoc tests. There were no statistical differences among groups. T0 = PND30; T1 = PND40; CD = Control Diet; HFD = High Fat Diet. (PDF) [file pone.0315197.s001.pdf]

Supporting Information

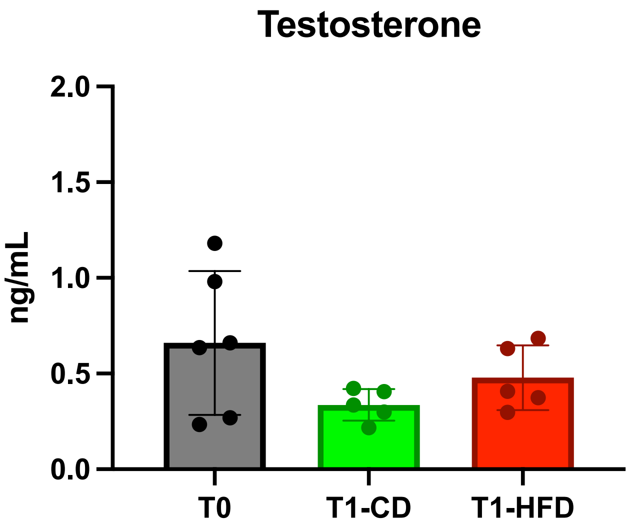

Supplemental Figure 1. **Testosterone levels during prepuberty.** Values are means ± SEMs (*n* = 5-6 rats per group). ANOVA analyses were performed followed by Tukey´s post hoc tests. There were no statistical differences among groups. T0 = PND30; T1 = PND40; CD = Control Diet; HFD = High Fat Diet.
