# Supplementary figures and images for: The type of diet consumed during prepuberty modulates plasma cholesterol, hepatic LXRα expression, and DNA methylation and hydroxymethylation during adulthood in male rats
Source: PLoS One. 2025 Jan 24;20(1):e0315197. doi: 10.1371/journal.pone.0315197 (PMC11761095; doi:10.1371/journal.pone.0315197)

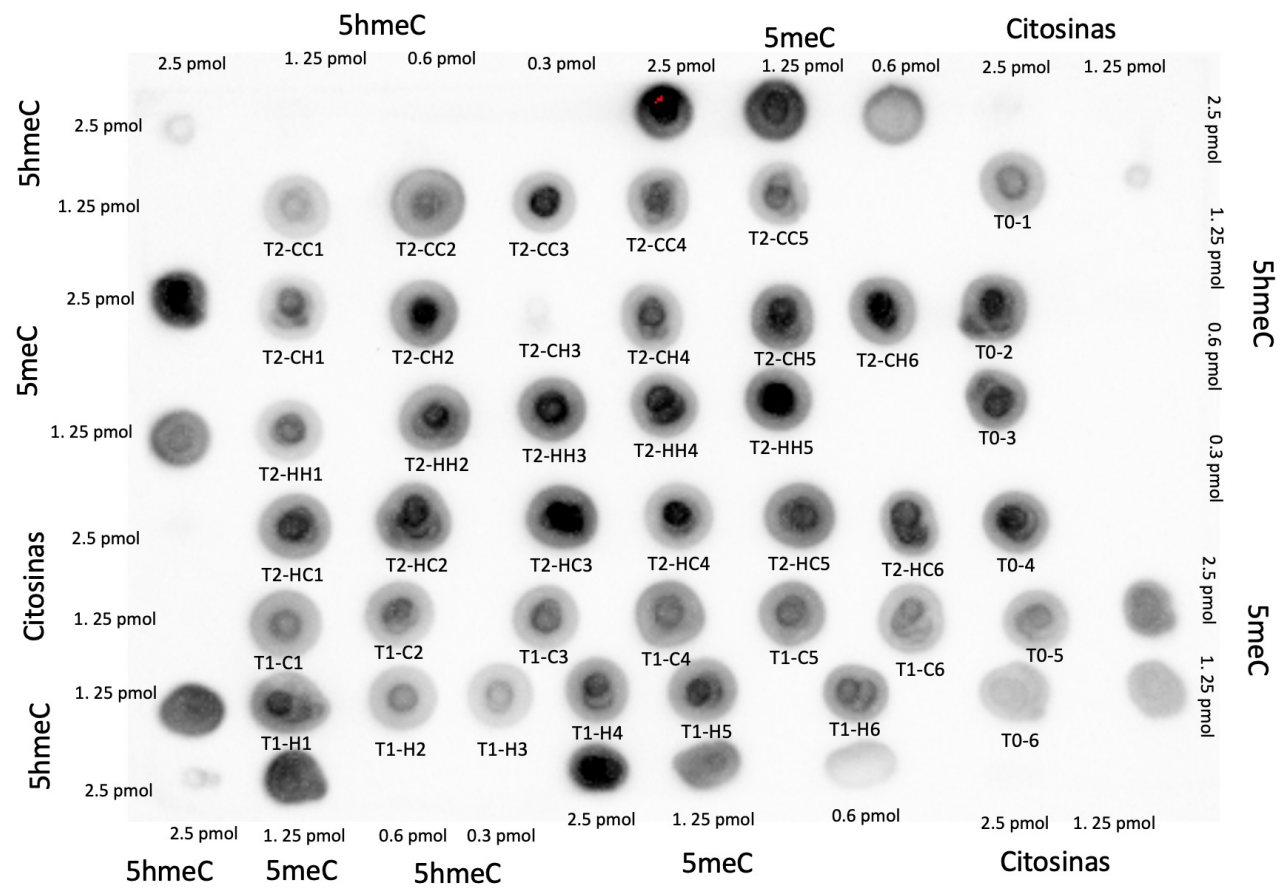

Figure 6A

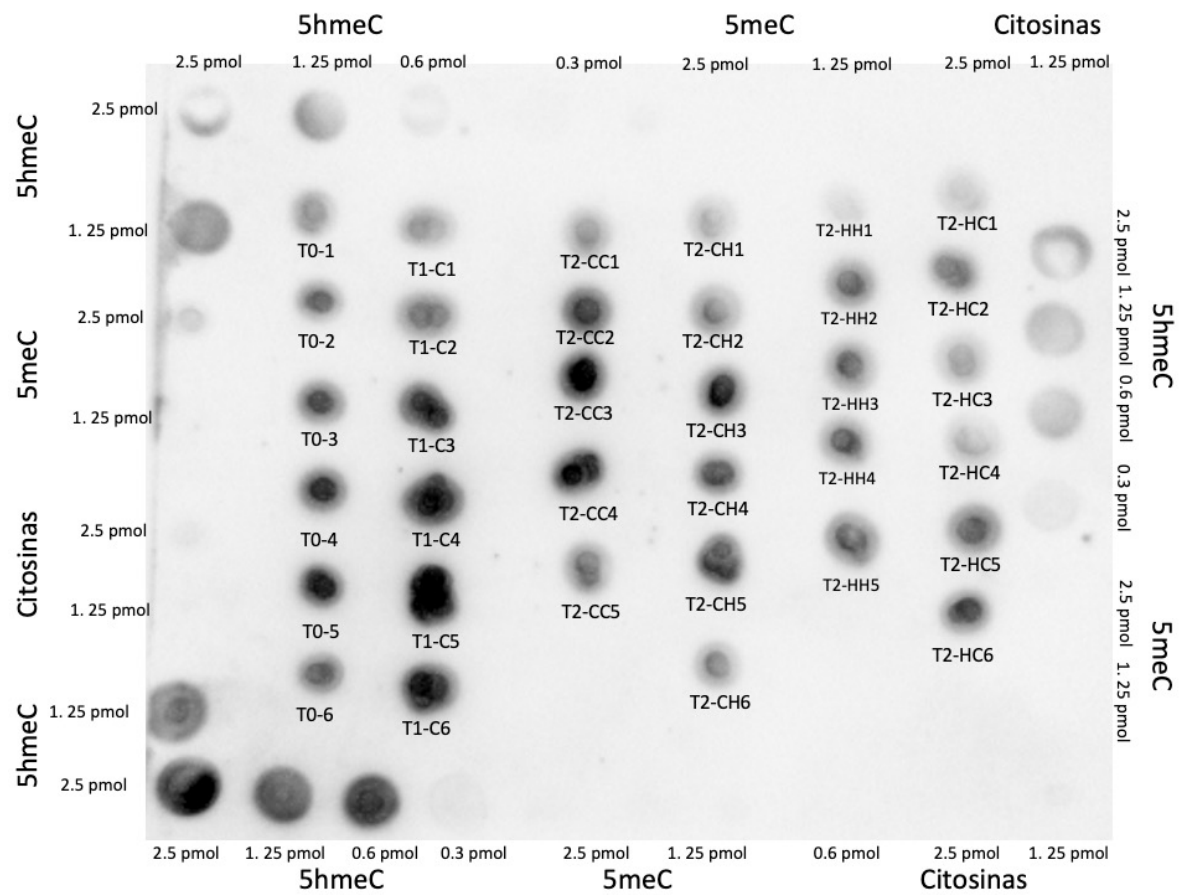

Figure 6B

Supplement: S2 Fig — Uncropped images for dot blots in Fig 6. (PDF) [file pone.0315197.s002.pdf]
