# Supplementary material for: The type of diet consumed during prepuberty modulates plasma cholesterol, hepatic LXRα expression, and DNA methylation and hydroxymethylation during adulthood in male rats
Source: PLoS One. 2025 Jan 24;20(1):e0315197. doi: 10.1371/journal.pone.0315197 (PMC11761095; doi:10.1371/journal.pone.0315197)
Supplement: S1 Table — (DOCX) [file pone.0315197.s003.docx]

**Supporting Information**

**Supplemental Table 1. List of primer sequences for genes analyzed in this study.**

Gene Forward Sequence Reverse sequence

*Actn*  GCTCTCTTCCAGCCTTCCTT AGTACTTGCGCTCAGGAGGA

*Hprt* CTGGTGAAAAGGACCTCTCG GGCCACATCAACAGGACTCT

*Srebf2* ACAGCCAGTTACCATCCAGC CAGCGTGGTCAAAACAAGGG

*Hmgcr* GAGCTTGCTGTGAGAACGTG ACCTCCACCAAGACTGATCG

*Ldlr* ACCGCCATGAGGTACGTAAG CGGCGCTGTAGATCTTTCTC

*Lxrα* CCACTTTACTGAGCTGGCCA GTTGTACCTCCGCGATGTCT

*Cyp7a1* TTGATTCCGTACCTGGGCTG CTGTGTCCAAATGCCTTCGC

*Actn* = actin*; Hprt* = hypoxanthine phosphoribosyltransferase 1; *Srebf2* = sterol regulatory element binding transcription factor 1; *Hmgcr* = 3-Hydroxy-3-Methylglutaryl-CoA reductase; *Ldlr* = Low Density Lipoprotein receptor; *Lxrα*  = liver X receptor-alpha; *Cyp7a1* = cytochrome P450 family 7 subfamily A member 1.
