# Supplementary material for: The type of diet consumed during prepuberty modulates plasma cholesterol, hepatic LXRα expression, and DNA methylation and hydroxymethylation during adulthood in male rats
Source: PLoS One. 2025 Jan 24;20(1):e0315197. doi: 10.1371/journal.pone.0315197 (PMC11761095; doi:10.1371/journal.pone.0315197)
Supplement: Attachment — Submitted filename: 240904_Response to reviewers.docx [file pone.0315197.s004.docx]

Víctor Sánchez-Margalet

Academic Editor

PLOS ONE

Mexico City, September 6^th^, 2024

PONE-D-24-19131

Dear Dr. Sánchez-Margalet (Víctor),

We appreciate the reviewers’ thoughtful comments and valuable contributions that have enabled to improve our manuscript titled “The Type of Diet Consumed During Prepuberty Modulates Plasma Cholesterol, Hepatic LXRα Expression, and DNA Methylation and Hydroxymethylation during Adulthood in Male Rats”. Below, please find our point-by-point response to the reviewers. We have highlighted in yellow the changes made in the revised manuscript.

**Reviewer 1:**

“The authors investigate prepuberty diets influence plasma cholesterol, hepatic LXR

expression, and DNA methylation and hydroxymethylation in male rats.

We thank the reviewer for their time invested in revising our manuscript and for their valuable suggestions. A detailed point-by-point answer to their comments is provided below.

**Comment 1: “**In the introduction the age of puberty for male rats should be described.”

**Response:** We have included the age corresponding to prepuberty in male rats in line 52.

**Comment 2:** “Line 40: what tissues?”

**Response:** We have included in lines 39-40 that the mentioned study used peripheral blood cells.

**Comment 3:** “Line 41: how does Igf2 alter metabolic flexibility”

**Response:** We have revised the manuscript to specify how IGF2 alters metabolic flexibility in line 42.

**Comment 4:** “Line 42: tends to be decreased - tends or is significantly decreased? ie is decreased”

**Response:** We have clarified that metabolic flexibility is decreased during obesity and high-fat overfeeding in line 43.

**Comment 5:** “Line 46: reference”

**Response:** We have revised the manuscript to include the corresponding reference now in line 47.

**Comment 6: “**Line 49-what hepatic genes?”

**Response:** We have included the hepatic genes that showed modified expression in the mentioned study in line 50-51.

**Comment 7:** “Line 50-what sex?”

**Response:** We have indicated that the results are from male rats in line 52.

**Comment 8:** “Line 73-what specific aspects of metabolic phenotype”

**Response:** We have included the specific aspects of the metabolic phenotype that were evaluated in line 75-76.

**Comment 9:** “All of the data (apart from histological and dot blot data) in figure 3-6 should be in bar graph with scatter plots”

**Response:** We have generated bar graphs with scatter plots for the suggested figures and made them available in Figshare, along with all the data included in this manuscript. However, we consider that our current figures are better suited to illustrate the effect over time, from the beginning of prepuberty to adulthood.

**Comment 10:** “Line 333 reference”

**Response:** We have included the corresponding reference in line 324.

**Comment 11:** “Line 335 define age of weaning”

**Response:** We have updated the manuscript to include the age of weaning in line 328.

**Comment 12:** “In the discussion, there should be some mention of any potential sex differences. Even though only males were studied in this project do the authors predict sex differences.”

**Response:** We have revised the manuscript to discuss potential sex differences.

**Reviewer 2:**

“This work brings a novelty to an important window of opportunity in development - prepuberty. Also, the study of HFD, that is widely used and studied, and it is well known to lead to chronic disease development throughout life. This paper is well-designed and described, addressing a great topic. However, some topics should be revised to better clarify the results and to be well understood, and then published.”

We thank this reviewer for their time invested in revising our manuscript and for his/her positive comments and valuable suggestions. A detailed point-by-point answer to their comments is provided below.

**Comment 1:** “In the introduction section. Second paragraph: when the gene is mentioned, it should be written in lowercase (Igf2), if it is a protein then uppercase (IGF2). Please review if it was correct.”

**Response:** We have reviewed the manuscript and have used lower case italics for gene names and uppercase for protein names. In the particular case of IGF2 in line 42, we are indeed referring to the protein**.**

**Comment 2**: “In the introduction section. Second paragraph: is it really necessary to mention other types of diet instead of focusing only on the HFD which was the diet studied? References 20, 21, 22, 23, 24, and 25.”

**Response:** We agree with the reviewer that the manuscript introduction should focus solely on HFD. However, there is a limited number of studies that evaluate the postnatal effects of the type of diet during critical windows of development on adult DNA methylation patterns and/or phenotype. Therefore, we believe it is important to maintain these references in our introduction to highlight that the type of diet during critical windows of development can influence the adult phenotype.

**Comment 3:** “The study evaluated only male rats. Nowadays, we know that sex has an isolated effect on some metabolic results. When it is not possible to assess females either, it could be taken into consideration to discuss it in the appropriate section.”

**Response:** We completely agree with the reviewer. We have revised the manuscript to include a discussion on potential sex differences and how they may impact the metabolic results observed in our model.

**Comment 4**: “For statistical analyses, were there outliers removed? And the variables had normal distribution? This information could be placed in the section 2.8.”

**Response:** We apologize for the omission of this information. We have revised the statistical analyses section to clarify these points.

**Comment 5**: “The meaning of “ * ” is not described in the legend of the figure.”

**Response:** We have included “*”meaning in the figure legends.

**Comment 6**: “It would be better if the group’s names were standardized (in text and figures are different from slopes).”

**Response:** We apologize for the oversight. We have modified the figures to standardize groups´ names in text and figures**.**

**Comment 7**: “The description of the results could be more concise, avoiding unnecessary mentions, because this could lead the reader to some misunderstanding. The results are confusing, maybe it would benefit from a rewrite. For example, in 3.2: “Upon observing that a CD during prepuberty decreased fat mass and increased lean mass in adulthood despite a chronic HFD feeding”. It was not what is shown in the graph, the effect is despite chronic HFD when compared to HH. HFD→C seems to be similar to CC. Is this correct? So, if it is misunderstood maybe a rewrite could solve it.”

**Response:** We agree with the reviewer and appreciate their observation. We have revised and updated the results to be more concise and clear, and we have included the appropriate references were necessary.

**Comment 8**: “All the abbreviations in the graphs must be in the legend.”

**Response:** We have revised and updated the figure legends to include all abbreviations.

**Comment 9**: “Would be more appropriate to establish the comparisons in the results? Such as in the 3.5 section “Based on the lower plasma and hepatic cholesterol concentrations observed in the C→HFD group during adulthood” this means that is compared to all other groups?”

**Response:** We apologize for the oversight. We have updated the manuscript to address this issue on lines 267-268.

**Comment 10:** “If it is not statistically significant then I believe it is not right to state any conclusion. In the 3.5 section is said: “Now, for Cyp7a1, there was a trend that followed the same pattern of expression than Lxrα, where CC and C🡪HFD groups had higher Cyp7a1 expression than HH and HFD🡪C (Fig. 5D-E).”. Again, in lines 308-310. However, in the figure, there are no signals indicating it. Or maybe it is missing in the figures.”

**Response:** We apologize for the confusion. We have revised the text to remove any conclusions that were not supported by statistical significance.

**Comment 11:** “Across the results section are discussed hypotheses but without references. Would be more appropriate if those parts of the texts were moved to the discussion section? Then the results would be more concise and the discussion more robust. OR merge the two sections (results and discussion).”

**Response:** We apologize for the omission and appreciate the reviewer´s suggestion. We have included the appropriate references in the results section. Additionally, we have revised the manuscript to ensure a clear distinction between the presentation of results and their interpretation.

**Comment 12**: “The sentence in lines 337-339 seems to be a little confusing, could the authors rewrite it for a better understanding?”

**Response:** We have rewritten the sentence now in line 330-331 for better understanding.

**Comment 13:** “In line 408, the abbreviation.”

**Response:** We have included the missing definition now in line 401.

**Comment 14:** “It is taken as a limitation the absence of other analyses in different tissues. Why is that missing in this paper? The results brought here can themself be explained and discussed, so perhaps it is not a limitation, but a future aim, some to be additional”

**Response:** We agree with the reviewer and have updated the discussion to frame the analysis of other tissues as a direction for future studies rather than as a limitation.

We hope that this new version will satisfy the reviewers and qualify our work for publication in PLOS One.

We look forward to hearing from you,

Sincerely,

Dr. Lilia Noriega, PhD.

Medical Sciences Researcher D

Department of Nutritional Physiology

National Institute of Medical and Nutritional Sciences “Salvador Zubirán”

Ph. +52 (55) 54870900 ext. 2802
