# Supplementary material for: The type of diet consumed during prepuberty modulates plasma cholesterol, hepatic LXRα expression, and DNA methylation and hydroxymethylation during adulthood in male rats
Source: PLoS One. 2025 Jan 24;20(1):e0315197. doi: 10.1371/journal.pone.0315197 (PMC11761095; doi:10.1371/journal.pone.0315197)
Supplement: Attachment — Submitted filename: 241031_Response to reviewers.docx [file pone.0315197.s005.docx]

Christopher Torrens

Academic Editor

PLOS ONE

Mexico City, October 31^th^, 2024

PONE-D-24-19131R1

Dear Dr. Torrens (Christopher),

We appreciate the reviewers’ thoughtful comments and valuable contributions that have enabled to improve our manuscript titled “The Type of Diet Consumed During Prepuberty Modulates Plasma Cholesterol, Hepatic LXRα Expression, and DNA Methylation and Hydroxymethylation during Adulthood in Male Rats”. Below, please find our point-by-point response to the reviewers. We have highlighted in yellow the changes made in the revised manuscript.

**Reviewer 3:** “: The study by Aguilar-Lozano et al explores the impact of diet around the time of puberty on adult metabolic health in rats. In this study, the authors specifically explore the association of feeding male rats either a control diet or a high fat diet between days 30 and 40 of age. The males are then either maintained on the same diet, or switched to the alternative diet, resulting in 4 groups. The specific aim was to see if a control diet around the time of puberty would prevent the development of metabolic disease in later life. In particular, the authors have focused on investigating hepatic function, histology, gene expression and DNA methylation.

On the whole, the manuscript is well-presented and well-written. However, I do have three main comments that need addressing.”

We thank the reviewer for their time invested in revising our manuscript and for their valuable suggestions. A detailed point-by-point answer to their comments is provided below.

**Comment 1:** “The first is that the diets appear to have different levels of vitamins, minerals and 1-Carbon metabolites and carriers such as choline. As the authors are feeding these diets to their rats for up to 180 days, would they anticipate that the differences in 5mC and 5hmC are due, in part, to the differing levels of 1-Carbon metabolites within their diets? Also, do the rats eat the same weight of the high fat diet per day as the control diet. Again, as the diets are not balanced for the micro-nutrient levels, if the rats consume a different weight of the diets, then would they be consuming differing levels of 1-Carbon metabolites and so be affecting their 5mC and 5hmC levels. As such, are the differences that are reported not due to the high levels of fat, per se, but just due to the different micro-nutrient compositions. This possibility needs to be addressed within the discussion.”

**Response:**

Food intake in grams decreases in high-fat diet (HFD) groups due to the diet´s higher energy density. As a result, we adjusted the vitamin, mineral, L-cystine, and choline content in HFD diets, as is commonly done in studies using AIN93M as a control diet, to account for reduced food intake. While we cannot fully rule out the influence of dietary 1-carbon metabolites on 5mC and 5hmC levels without specific assessments, we have controlled for this to the extent possible by balancing these micronutrients in relation to intake.

However, we recognize that a direct assessment of 1-carbon metabolites in plasma and liver could provide further insight into any differences in 5mC and 5hmC levels. We have addressed this issue into the discussion on line 420 to 426.

**Comment 2:** “My second question is, how much variability in the start of puberty is there between the males? As the authors have started all the males on their diets from 30 days of age, could some males be closer to puberty than others and so could they be affected differently when placed on their respective diet? Could the authors have defined their pubertal status in a more precise way through hormonal assessment?

**Response:**

To account for variability in pubertal timing, we measured testosterone levels at postnatal day 30 (T0) and postnatal day 40 (T1) in both diet groups to confirm that all animals were still in the prepubertal stage. The prepubertal stage typically corresponds to testosterone concentrations bellow 2.1 ng/mL (Lee et al). In our study, testosterone levels averaged 0.336 ng/mL (95% CI: 0.233 to 0.439), and no significant variability was observed among subjects within the same group. This assessment confirmed that none of the animals had reached puberty by these time points. Although natural biological variability exists, our hormonal assessment provided a reliable indicator of prepubertal status. We have added this information to the methods section at line 92.

Lee VW, de Kretser DM, Hudson B, Wang C. Variations in serum FSH, LH and testosterone levels in male rats from birth to sexual maturity. J Reprod Fertil. 1975 Jan;42(1):121-6. doi: 10.1530/jrf.0.0420121. PMID: 1110463.

**Comment 3:** “My final comment relates to Figure 6. I find this figure a little confusing as it is not clear which group is represented at T0 in each of the dot blots. Also, does T0 equate to the start of the dietary challenges (i.e. 30 days of age), and so T1 is 40 days and T2 210 days? If so, why not use the days as T0, T1 and T2 are not used elsewhere in the manuscript. On the dot blot for 5hmC, should 'T1-C' be 'T1-CC'? Finally, I find it interesting that there is a significant increase in 5hmC in the CC and C-HFD groups at day 40, but no difference in levels of 5mC at the same time in the same animals. Would the authors anticipate that a significant increase in 5hmC would suggest a shift towards a reduction in 5mC levels as 5hmC is an intermediate state in a shift towards demethylation? I would welcome the authors comments on the lack of connection.

**Response:**

We apologize for any confusion. We have clarified the meaning of T0, T1, and T2 in the figure legends for Figures 1, 4 and 6. T0, T1, and T2 represent the time points at which measurements were performed, specifically:

- T0 = Postnatal day (PND) 30,
- T1 = PND 40,
- T2 = PND 210.

In other figures, we opted to display postnatal days on the y-axis for clarity. In the dot blot, however, we used the combination of the time point (T0, T1, T2) and the diet group to provide specific context for each group´s timing and diet.

Regarding the labeling issue in the dot blot, we have corrected “T1-C” to “T1-CD” to align with the description in Figure 1A and the methods section, where “CC” refers to control diet throughout the study. Thus, "T1-CD" specifically represents the control diet group at day 40.

Concerning the increase in 5hmC levels without an associated decrease in 5mC, it is important to note that we assessed global rather than site-specific DNA methylation and hydroxymethylation. Thus, while 5hmC serves as an intermediate in demethylation, this does not necessarily lead to a direct inverse relationship with 5mC on a global level. The majority of CpG sites in the genome remain methylated, whereas hydroxymethylation is typically enriched in regulatory regions of actively transcribed genes. Based on previous findings (Reizel et al., reference 19), we expected that hydroxymethylation could increase as part of the demethylation process, though global 5mC levels might remain stable. We have incorporated this explanation into the discussion on line 396 and included references from Charlton et al. (reference 57) and Ivanov et al. (reference 53) to support this interpretation.

We hope that this new version will satisfy the reviewer and qualify our work for publication in PLOS One.

We look forward to hearing from you,

Sincerely,

Dr. Lilia Noriega, PhD.

Medical Sciences Researcher D

Department of Nutritional Physiology

National Institute of Medical and Nutritional Sciences “Salvador Zubirán”

Ph. +52 (55) 54870900 ext. 2802
